# PATIENTS' ATTITUDE TOWARDS MEDICAL STUDENTS ROTATING IN THE DERMATOLOGY CLINIC

**DOI:** 10.4103/0019-5154.39734

**Published:** 2008

**Authors:** Iqbal Bukhari, Omar AlAkloby, Wafa Al Saeed

**Affiliations:** *From Dermatology Department, Community Medicine Department, College of Medicine, King Faisal University, Dammam, Saudi Arabia*

**Keywords:** *Attitudes*, *dermatology subspeciality*, *education*, *medical student*

## Abstract

**Objective:**

To study the attitudes of the patients towards medical students rotating in the dermatology clinic in the King Fahad Hospital of the University (KFHU).

**Materials and Methods:**

One hundred and two adult outpatients attending the KFHU in Alkhobar, Saudi Arabia during the period March to June 2004 completed a questionnaire to evaluate their receptiveness towards medical students attending with the dermatologist.

**Results:**

Almost 57% preferred physician and medical student participation in their care and 46% welcomed their presence during physical examination. The majority of patients (64.8%) felt comfortable disclosing personal information to the medical student and (68.7%) enjoyed the interaction with the medical students. Patients (63.7%) agreed that the students understood their healthcare needs.

**Conclusion:**

The majority of the patients in this study enjoyed their interactions with the students and felt comfortable disclosing information. Some patients want to spend time alone with the physician so permission for medical student participation should be requested.

## Introduction

Outpatient clinical medicine is considered of crucial importance in teaching clinical medicine in ambulatory settings. The medical college curriculum of King Faisal University requires rotating of the medical students in the subspeciality clinics during the fifth year of the medical school which is carried out at the King Fahad Hospital of the University (KFHU).^1^ Medical students are generally accepted by patients in the outpatient clinics at KFHU but the patients’ receptiveness of medical students has not been studied in Saudi Arabia. Interestingly, attitudes towards medical students have been found favorable by some researchers in dermatology, internal medicine and surgery clinics.^2^–^4^ In this study we try to examine the patients’ attitude towards medical students at the dermatology clinics in KFHU.

## Materials and Methods

Departmental approval for this study was pursued. Only patients whom the medical students participated in their management completed an anonymous survey at the end of their visit at the dermatology clinic. The study protocol was requested from Dr. Jeffrey Miller - he and his colleagues had conducted a similar study.^2^ The survey consisted of 15 questions asking about patient demographic information and preferences regarding medical student participation. The data was collected by the fifth year medical students during their rotation in the dermatology department. Usually, fifth-year medical students rotate through the clinic for three weeks and often see the patients with the attending physician. The medical student introduces himself or herself to the patient and then takes a history but the performance of the physical examination is done in the presence of the attending physician. The student then discusses the findings with the attending physician. Validation of the questions was done by three dermatology consultants while reliability was accomplished by taking a sample of 10 patients and repeating the same questions. Statistical analysis of the patients'survey included two-way frequency tables with chi-square analysis to compare distributions of categorical responses across the levels of the demographic variables. The average of four receptiveness items on the survey that used the Likert scale was used as overall receptiveness score. Values between 1 and 5 with lower numbers indicate higher receptiveness to the students. The comparison of the mean response of Likert scale across the levels of demographic variables was done by analysis of variance (ANOVA). All analyses were carried out with use of SPSS Version 12.0 statistical software package.

## Results

One hundred and two patients completed the survey. Demographic data are shown in Table 1. The results of the patients’ preferences regarding medical student participation in the outpatient dermatology clinic and their participation in the physical examination are listed in Table 2. This showed that the majority of patients preferred the student with the physician (56.9%) or had no preference (23.5%). Those who preferred to see the physician alone composed 19%. Student participation in the physical examination showed almost similar results. Patient responses to a variety of patient preference questions are listed in Table 3. As noted from that table, 51% of patients wanted time alone with the physician; 64.8% felt comfortable disclosing personal information to the student; whereas 22.9% were uncomfortable. The majority of patients enjoyed medical student interaction (94.2%) and felt medical students understood their healthcare needs (68.7%). A total of 22.8% of patients did not feel they received more attention with medical student participation. Results of chi-square showed that there is significant statistical difference between younger and older patients regarding participation of medical students in their care. Older patients (>30 years) more likely preferred to have students with the physician than younger patients (crosstable). No statistical difference was found between the sex and level of education. Regarding preference of physical examination, no difference was found between the age groups, sex or level of education and previous number of visits. Patients who were 30 years old or older were more likely to prefer a student with a physician in their care with no sex or level of education differences. Results of the ANOVA of receptiveness items showed significant association of preference of same sex for physical examination. T-test showed that males were more (2.64) to prefer having same sex than females (*P* = 0.001). Males had higher score than females. T-test and ANOVA of receptiveness items showed no significant differences between age groups, sex, education level and previous number of visits. Crosstable (chi-square) of the total attitude score with demographic data showed there were no significant statistical differences between the total attitude scores and the demographic data (age, sex, education level and previous number of visits).

**Table 1:** Demographics

Age (years)	
<30	48 (47.1)
>30	54 (52.9)
Sex	
Male	22 (21.6)
Female	80 (78.4)
Education	
Preparatory and lower	38 (37.3)
Secondary	30 (29.4)
Technical school	7 (6.9)
College graduate	24 (23.5)
Postgraduate	3 (2.9)
Previous visits with medical students	
1-3	39 (38.2)
4-5	34 (33.3)
>5	21 (20.6)

Figures given in parenthesis indicates percentage

**Table 2:** Distribution of patient preference response

Patient preference	Frequency	Percentage
Medical student participation		
Physician alone	20	19
Student with physician	58	56.9
No preference	24	23.5
Physical examination		
Physician alone	33	32.4
Student with physician	47	46.1
No preference	22	21.6

**Table 3:** Distribution of patient responses

Survey questions	Strongly agree	Agree	Undecided	Disagree	Strongly disagree
Prefer same sex	57 (55.9)	21 (20.6)	13 (12.7)	2 (2)	9 (8.8)
Wanted time alone	36 (35.3)	16 (15.7)	17 (16.7)	4 (3.4)	29 (28.4)
With the physician					
Felt comfortable	33 (32.4)	33 (32.4)	13 (12.7)	8 (7.8)	15 (14.7)
Disclosing personal information					
Enjoyed interacting	42 (41.2)	28 (27.5)	17 (16.7)	4 (3.9)	11 (10.8)
With the medical student					
Received more attention	35 (34.3)	22 (21.6)	22 (21.6)	6 (5.9)	17 (16.7)
Medical student					
Understood patient's	36 (35.3)	29 (28.4)	30 (29.4)	1 (1)	6 (5.9)
health need					

Figures given in parenthesis indicates percentage

## Discussion

The majority of patients had favorable attitudes about their interactions with the medical students. A total of 57% of patients enjoyed their interaction with the students and 19% preferred to see the physician alone. More than 64% of patients were comfortable disclosing personal information to the students and 63.7% felt that the students understood their healthcare needs while 68.7% enjoyed interacting with the medical students. However, because of the cultural and religious nature of Saudi women we expected them to have negative attitude towards medical student participation in their care. It is very interesting that despite the high percentage of females in this study (78.4%) their responses were on the positive side. Many patients commented that the involvement of medical students in the outpatient care is a good training for them and a great opportunity to learn and gain experience with patients.

Even though the responses of our patients mentioned above were positive, they were less than that in the Townsend *et al.* report.^2^ This may be attributed to differences in culture, education and sex, especially that the majority of our study population were females (78.4%), whereas in that study the percentage of females (55.3%) is close to the percentage of males (44.7%). 51% of the patients wanted time alone with the physician, 22.5% of the patients did not feel comfortable disclosing personal information to the medical student and 55.9% of the patients would prefer a student of the same sex to perform the physical examination. However, comparing our results with the Townsend *et al.* study^2^ we did not find a significant correlation between patients’ preferences and their demographic information. In conclusion medical student participation in outpatient clinics is an integral component of their medical school education and the patient is often the focal point of the teaching process. In our university-based dermatology clinic, most patients enjoy medical student participation in their care. However, identifying patients uncomfortable disclosing personal information to the medical student and patients wanting time alone with the physician are equally important. To our knowledge this is the first study of such type done in our area and more similar studies should be conducted at the centers in each medical specialty to better evaluate the patients’ perceptiveness towards medical students.

Some of the points that were suggested by the patients include: same sex is preferable in patient care, the physician should be the one questioning the patient about his illness, decrease the number of students per session, physician alone should examine the genital areas, provide more privacy for the patient and informed consent regarding medical student participation should be part of the patient-medical student-physician encounter.
